# Dietary Fat and Prostate Cancer Relationship Using Trimmed Regression Under Uncertainty

**DOI:** 10.3389/fnut.2022.799375

**Published:** 2022-03-10

**Authors:** Muhammad Aslam, Ali Hussein AL-Marshadi

**Affiliations:** Department of Statistics, Faculty of Science, King Abdulaziz University, Jeddah, Saudi Arabia

**Keywords:** regression, correlation, classical statistics, neutrosophic statistics, cancer data analysis

## Abstract

In this paper, a new trimmed regression model under the neutrosophic environment is introduced. The mathematical model of the new regression model along with its neutrosophic form is given. The methods to find the error sum of square and trended values are also given. The trimmed neutrosophic correlation is also introduced in the paper. The proposed trimmed regression is applied to prostate cancer. From the analysis, it is concluded that the proposed model provides the minimum error sum of square as compared to the existing regression model under neutrosophic statistics. It is found that the proposed model is quite effective to forecast prostate cancer patients under an indeterminacy setting.

## Introduction

The regression analysis has been applied to study the relationship between two variables that are correlated in a variety of fields such as business, medical science, and weather forecasting. One of the main objectives of the regression model is the forecasting of the response variable using the information of the independent variable. For example, the relationship between prostate cancer and dietary fat can be studied using the regression model. On the other hand, the correlation analysis is done to see the degree of relationship between two correlated variables. In the regression models, a mode is selected which has the minimum error sum of square. Abdul-Wahab et al. (1–4) discussed the applications of the regression models in various fields.

Prostate cancer kills around 34,130 people in the USA every year where more than 248,530 patients are coming every year (https://www.cancer.org/cancer/prostate-cancer/about/key-statistics.html). Smoking is one of the main reasons for this type of cancer and patient has to bear a costly treatment of this disease. Jemal et al. (5) studied the relationship between age and prostate cancer. Rahib et al. (6) expected an increase in deaths due to cancer. Arnold et al. (7) found a strong relationship between smoking and cancer. Torre et al. (8) studied prostate cancer patients in the UK. According to Lin et al. (9), cancer patients remain uncomfortable during the rest of life. Siegel et al. (10) reported the yearly deaths due to cancer in the USA. Prostate cancer is very common in men and can be controlled if it is diagnosed at an early stage. According to Cao et al. (11) “Prostate cancer is the third highest cause of male mortality in the developed world.” Lin et al. (9) pointed out the high death rate due to prostate cancer. Scarton et al. (11, 12) studied various factors that cause cancer. Applegate et al. (13) presented a study on the relationship between soy food and cancer. Aslam and Albassam (14) studied the relationship between prostate cancer and dietary fat. More information can be seen in (15).

As mentioned earlier, the regression models are applied for the purpose of the estimation and forecasting of different fields. The presences of the extreme values in the data affect the forecasting and estimation significantly. Several methods are applied to remove these extreme or outliers from the data for a better analysis of the data at hand. The idea of a trimmed average is applied to calculate the average of the observations after removing a specific percentage of the extreme values from the data. The use of the trimmed method is helpful in minimizing the variation in the data. Oten and de Figueiredo (16–22) presented various trimmed methods in various fields.

The regression models and trimmed mean are applied under the assumption that there is no uncertain observation in the data. In practice, as mentioned by (23), the observations can be uncertain, imprecise, and in the interval. For this kind of data, the existing regression model and trimmed mean cannot be applied. To deal with this type of data, statistical methods developed under fuzzy logic are applied. The fuzzy logic-based statistical methods provide the results in an uncertain environment. Saritas et al. (24–28) analyzed prostate cancer data using fuzzy logic. Vela-Rincón et al. (29) presented the idea of a trimmed average under the fuzzy logic.

Smarandache (30) gave the idea of neutrosophic statistics and declared it as the generalization of classical statistics. Neutrosophic statistics has advantages over classical statistics as it gives additional information about the measure of indeterminacy. The neutrosophic statistics is applied when the data is vogue, unclear, imprecise, and indeterminate, Chen et al. (31–35).

As mentioned before that the idea of a trimmed average is applied to remove the extreme values from the data. By exploring the literature and according to the best of our knowledge, there is no work on trimmed regression under neutrosophic statistics. In this article, we will present the trimmed neutrosophic regression originally. We will present the trimmed neutrosophic regression, trended values, and error sum of square in the indeterminate environment. The application of the proposed regression model is given in prostate cancer data. It is expected that the proposed regression will be efficient than the existing regression model. In addition, it is expected that the proposed model will be quite effective to be applied for forecasting and prediction of prostate cancer.

## The Proposed Regression Model

Suppose that we have *n*_*N*_ ϵ [*n*_*L*_, *n*_*U*_] pairs of observations as (*x*_1*N*_, *y*_1*N*_), …, (*x*_*nN*_, *y*_*nN*_), where *x*_*nN*_ is an independent variable and *y*_*nN*_ be a dependent variable. It is assumed that both neutrosophic variables are correlated. For the implementation of the proposed trimmed regression model, the neutrosophic data is arranged in ascending order in variable *x*_*nN*_ or *y*_*nN*_. Let ω be the percentage of the removed values from both variables. For example, if ω = 6 and we arranged the data in ascending according to the variable *x*_*nN*_, it means that three pairs of observations will be removed from the starting and three pairs of observations will be removed from the end of the data. Using this information, the trimmed regression model under neutrosophic statistics, say *Y*_*NT*_ ϵ [*Y*_*LT*_, *Y*_*UT*_] is given as


(1) $$\begin{array}{l} Y_{NT}=a_{NT}+b_{NT}X_{NT};a_{NT}\epsilon \left[a_{LT},a_{UT}\right],b_{NT}\epsilon \left[b_{LT},b_{UT}\right] \end{array}$$

where *a*_*NT*_ ϵ [*a*_*LT*_, *a*_*UT*_] and *b*_*NT*_ ϵ [*b*_*LT*_, *b*_*UT*_] are slope and rate of change of the proposed trimmed regression model.

The proposed trimmed regression in neutrosophic form can be written as


(2) $$\begin{array}{l} {Ŷ}_{NT}=\left(a_{LT}+a_{UT}I_{NaT}\right)+\left(b_{LT}+b_{UT}I_{NbT}\right)\left(X_{LT}+X_{UT}I_{NXT}\right);\\ I_{NaT}\epsilon \left[I_{LaT},I_{UaT}\right],I_{NbT}\epsilon \left[I_{LbT},I_{UbT}\right],I_{NXT}\epsilon \left[I_{LXT},I_{UT}\right] \end{array}$$

where *a*_*LT*_, *b*_*LT*_, *X*_*LT*_ be the lower values of indeterminate interval and known as determinate values under classical statistics. On the other hand,*a*_*UT*_*I*_*NaT*_; *I*_*NaT*_ ϵ [*I*_*LaT*_, *I*_*UaT*_], *b*_*UT*_*I*_*NbT*_; *I*_*NbT*_ ϵ [*I*_*LbT*_, *I*_*UbT*_], *X*_*UT*_*I*_*NXT*_; *I*_*NXT*_ ϵ [*I*_*LXT*_, *I*_*UT*_] are indeterminate values of indeterminate interval. The proposed trimmed regression model is a generalization of the regression model proposed by Aslam and Albassam (14). The operations of neutrosophic numbers can be seen in Chen et al. (31, 32). The trimmed neutrosophic correlation, say *r*_*NT*_ ϵ [*r*_*LT*_, *r*_*UT*_] is defined as


(3) $$\begin{array}{l} r_{NT}=\frac{n_{NT}\sum X_{NT}Y_{NT}-\;\sum X_{NT}\sum Y_{NT}}{\sqrt{\left\{n_{NT}\sum {\left(X_{NT}\right)}^{2}-{\left(\sum X_{NT}\right)}^{2}\right\}\left\{n_{NT}\sum {\left(Y_{NT}\right)}^{2}-{\left(\sum X_{NT}\right)}^{2}\right\}}} \end{array}$$

The neutrosophic form of *r*_*NT*_ ϵ [*r*_*LT*_, *r*_*UT*_] is given by


(4) $$\begin{array}{l} r_{NT}=r_{LT}+r_{UT}I_{NrT}{;I}_{NrT}\epsilon \left[I_{LrT},I_{UrT}\right] \end{array}$$

where *r*_*LT*_ is a determinate part and *r*_*UT*_*I*_*NrT*_; *I*_*NrT*_ ϵ [*I*_*LrT*_, *I*_*UrT*_] is an indeterminate part. The proposed trimmed neutrosophic correlation reduces to (14) if no pair of observations is trimmed from the data. The following steps can be applied to run the proposed regression model on real data.

1. Arrange the data of *X*_*N*_ or *Y*_*N*_ in ascending order.
2. Fix the trimmed value ω. Indicate ω extreme values in *X*_*N*_. Remove $\frac{\omega }{2}$ pair of (*X*_*N*_, *Y*_*N*_) from the start and $\frac{\omega }{2}$ pair from the end.
3. Fit the proposed regression on trimmed data.
4. Determine the neutrosophic trended values and neutrosophic error sum of square.

## Application for Prostate Cancer

In this section, the application of the proposed regression model is applied to the prostate cancer data of 30 countries. The present case study is based on two variables namely dietary fat and death rate. The decision-makers are interested to see the relationship between these two variables. For this study, dietary fat is considered as the independent variable, and the death rate is labeled as the dependent variable. Let *X*_*N*_ denote the variable dietary fat and *Y*_*N*_ denotes the death rate. The purpose of this study is to determine the effect of dietary effects on the death rate. The neutrosophic data of variables *X*_*N*_ and *Y*_*N*_ is selected from Aslam and Albassam (14) and shown in Table 1 for easy reference. From Table 1, it can be seen that the given data is given in indeterminate intervals; therefore, the classical regression model under classical statistics cannot be applied to study the relationship between death rate and dietary fat. Aslam and Albassam (14) presented the neutrosophic regression analysis for the same data. We now apply the idea of trimmed regression on the same data. The proposed regression analysis can be applied as follows

5. Arrange the data of *X*_*N*_ and *Y*_*N*_ in ascending order.
6. Fix the trimmed value ω = 3%. Indicate the six extreme values in *X*_*N*_. Remove three pairs of (*X*_*N*_, *Y*_*N*_) from the start and end three pairs of (*X*_*N*_, *Y*_*N*_) from the end.
7. Fit the proposed regression on this trimmed data.
8. Determine the neutrosophic trended values and neutrosophic error sum of square $\overset{n_{N}}{\underset{i=1}{\sum }}{\left(Y_{NT}-{Ŷ}_{NT}\;\right)}^{2}$

**Table 1:** Prostate cancer death rate of 30 countries.

**County No**.	**Diet Fat**	**D-rate**	**County No**.	**Diet Fat**	**D-rate**
1	(38,38)	(0.9,1.1)	16	(97,97)	(10.1,10.3)
2	(29,31)	(1.3,1.3)	17	(73,75)	(11.4,11.4)
3	(42,42)	(1.6,1.6)	18	(112,112)	(11.1,11.1)
4	(57,57)	(4.5,4.5)	19	(100,100)	(13.1,13.3)
5	(96,98)	(4.8,4.10)	20	(134,134)	(12.9,13.1)
6	(47,49)	(5.4,5.6)	21	(142,142)	(13.4,13.4)
7	(67,67)	(5.5,5.5)	22	(119,119)	(13.9,14.2)
8	(72,74)	(5.6,5.6)	23	(137,137)	(14.4,14.4)
9	(93,93)	(6.4,6.6)	24	(152,152)	(14.4,14.6)
10	(58,58)	(7.8,7.8)	25	(129,129)	(15.1,15.3)
11	(95,95)	(8.4,8.6)	26	(156,156)	(15.9,15.9)
12	(67,69)	(8.8,8.8)	27	(147,147)	(16.3,16.4)
13	(62,62)	(9,9)	28	(133,133)	(16.8,16.9)
14	(96,96)	(9.1,9.1)	29	(132,132)	(18.4,18.4)
15	(86,87)	(9.4,9.4)	30	(143,144)	(12.4,12.6)

The trimmed data of *X*_*NT*_ and *Y*_*NT*_ is shown in Table 2. The neutrosophic trimmed regression model using the data given in Table 2 is given by


(5) $$\begin{array}{l} {Ŷ}_{NT}=\left[0.2306,0.0567\right]+\left[0.1032,0.1049\right]X_{NT} \end{array}$$

**Table 2:** Trimmed Prostate cancer death rate of 30 countries.

**County No**.	**Diet Fat**	**D-rate**	**County No**.	**Diet Fat**	**D-rate**
1	(57,57)	(4.5,4.5)	13	(97,97)	(10.1,10.3)
2	(96,98)	(4.8,4.1)	14	(73,75)	(11.4,11.4)
3	(47,49)	(5.4,5.6)	15	(112,112)	(11.1,11.1)
4	(67,67)	(5.5,5.5)	16	(100,100)	(13.1,13.3)
5	(72,74)	(5.6,5.6)	17	(134,134)	(12.9,13.1)
6	(93,93)	(6.4,6.6)	18	(142,142)	(13.4,13.4)
7	(58,58)	(7.8,7.8)	19	(119,119)	(13.9,14.2)
8	(95,95)	(8.4,8.6)	20	(137,137)	(14.4,14.4)
9	(67,69)	(8.8,8.8)	21	(129,129)	(15.1,15.3)
10	(62,62)	(9,9)	22	(133,133)	(16.8,16.9)
11	(96,96)	(9.1,9.1)	23	(132,132)	(18.4,18.4)
12	(86,87)	(9.4,9.4)	24	(143,144)	(12.4,12.6)

The neutrosophic form of Ŷ_*NT*_ for the cancer data is given by


(6) $$\begin{array}{l} {Ŷ}_{NT}=\left(0.2306-0.0567I_{NaT}\right)+\left(0.1032+0.1049I_{NbT}\right)X_{NT};\\ I_{NaT}\epsilon \left[0,3.06\right],I_{NbT}\epsilon \left[0,0.02\;\right] \end{array}$$

The proposed regression model can be interpreted as when *X*_*NT*_ ϵ [0, 0], the death rate will be from 0.2306 and 0.0567. The rate of change in the death rate due to the dietary fat is from 0.1032 to 0.1049. The neutrosophic correlation between dietary fat and death rate is from 0.7996 and 0.7910. From this study, it can be noted that the proposed regression analysis provides the values of intercept and rate of change values in indeterminate intervals rather than the exact values as in regression under classical statistics. Therefore, the proposed regression analysis is quite effective to be applied to study the relationship between dietary fat and death rate under indeterminacy.

## Comparative Studies Based on Cancer Data

Aslam and Albassam (14) applied the neutrosophic regression model on the prostate cancer data. As mentioned earlier, in the regression theory, a regression model having the smaller values of the error sum of square is minimum is called an efficient regression model. We now compare the efficiency of the proposed regression model with Aslam and Albassam (14) regression model in terms of neutrosophic error sum of square $\overset{n_{NT}}{\underset{i=1}{\sum }}{\left(Y_{NT}-{Ŷ}_{NT}\right)}^{2}$, where *Y*_*NT*_ and Ŷ_*NT*_ are original values and trend values, respectively. The values of Ŷ_*NT*_ and $\overset{n_{NT}}{\underset{i=1}{\sum }}{\left(Y_{NT}-{Ŷ}_{NT}\right)}^{2}$ for both regression models are shown in Table 3. From Table 3, it can be seen that the values of $\overset{n_{NT}}{\underset{i=1}{\sum }}{\left(Y_{NT}-{Ŷ}_{NT}\right)}^{2}$ are smaller for the proposed regression model as compared to the existing values of $\overset{n_{NT}}{\underset{i=1}{\sum }}{\left(Y_{NT}-{Ŷ}_{NT}\right)}^{2}$. For example, the values of the error sum of square is $\overset{n_{NT}}{\underset{i=1}{\sum }}{\left(Y_{NT}-{Ŷ}_{NT}\right)}^{2}=\left[147.41,155.11\right]$ from the existing regression proposed by (14). The values of the error sum of square are $\overset{n_{NT}}{\underset{i=1}{\sum }}{\left(Y_{NT}-{Ŷ}_{NT}\right)}^{2}=\left[128.18,137.70\right]$ from the proposed regression model. By comparing the values of $\overset{n_{NT}}{\underset{i=1}{\sum }}{\left(Y_{NT}-{Ŷ}_{NT}\right)}^{2}$of both regressions, it is concluded that the proposed model is better than the existing model proposed by Aslam and Albassam (14). Therefore, the proposed model can be used for the forecasting of prostate cancer under the presence of uncertainty.

**Table 3:** The trended values and error sum of square for two regression models.

**Existing regression**		**Proposed regression**	
** ${\hat{Y}}_{N}$ **	** $\sum_{i=1}^{n_{N}}{\left(Y_{N}-{Ŷ}_{N}\right)}^{2}$ **	** ${\hat{Y}}_{N}$ **	** $\sum_{i=1}^{n_{N}}{\left(Y_{N}-{Ŷ}_{N}\right)}^{2}$ **
(3.15, 3.24)	(5.49, 4.24)		
(2.22, 2.35)	(0.85, 1.11)		
(3.69, 3.61)	(4.40, 4.07)		
(5.39, 5.34)	(0.80, 0.71)	(6.11, 6.04)	(2.59, 2.37)
(9.81, 10.05)	(25.19, 35.44)	(10.13, 10.34)	(28.47, 38.99)
(4.26, 4.42)	(1.28, 1.38)	(5.08, 5.20)	(0.10, 0.15)
(6.53, 6.49)	(1.06, 0.98)	(7.14, 7.09)	(2.70, 2.52)
(7.09, 7.29)	(2.24, 2.87)	(7.65, 7.82)	(4.24, 4.95)
(9.47, 9.47)	(9.48, 8.29)	(9.82, 9.81)	(11.73, 10.36)
(5.51, 5.45)	(5.23, 5.48)	(6.21, 6.14)	(2.51, 2.73)
(9.70, 9.70)	(1.70, 1.23)	(10.03, 10.02)	(2.66, 2.04)
(6.53, 6.72)	(5.14, 4.31)	(7.14, 7.30)	(2.74, 2.24)
(5.96, 5.91)	(9.21, 9.50)	(6.62, 6.56)	(5.62, 5.92)
(9.81, 9.82)	(0.51, 0.52)	(10.13, 10.13)	(1.07, 1.07)
(8.68, 8.78)	(0.51, 0.37)	(9.10, 9.18)	(0.08, 0.04)
(9.93, 9.93)	(0.02, 0.13)	(10.23, 10.23)	(0.01, 0.00)
(7.21, 7.41)	(17.54, 15.91)	(7.76, 7.93)	(13.22, 12.03)
(11.63, 11.66)	(0.28, 0.31)	(11.78, 11.81)	(0.47, 0.51)
(10.27, 10.28)	(7.99, 9.09)	(10.54, 10.55)	(6.50, 7.53)
(14.12, 14.19)	(1.50, 1.18)	(14.05, 14.12)	(1.33, 1.04)
(15.03, 15.10)	(2.66, 2.92)	(14.88, 14.88)	(2.19, 2.44)
(12.42, 12.46)	(2.17, 3.00)	(12.50, 12.54)	(1.93, 2.72)
(14.46, 14.53)	(0.00, 0.01)	(14.36, 14.43)	(0.00, 0.00)
(16.16, 16.25)	(3.12, 2.75)	(13.54, 13.59)	(2.43, 2.89)
(13.56, 13.61)	(2.37, 2.83)	(13.95, 14.01)	(8.10, 8.29)
(16.62, 16.71)	(0.51, 0.66)	(13.85, 13.91)	(20.69, 20.12)
(15.60, 15.68)	(0.48, 0.51)	(14.98, 15.17)	(6.68, 6.62)
(14.01, 14.07)	(7.76, 7.97)		
(13.90, 13.96)	(20.24, 19.71)		
(15.14, 15.33)	(7.54, 7.50)		
Sum	[147.41, 155.11]		[128.18, 137.70]

## Comparison in Trended Values Based on Cancer Data

**Figure 1:**
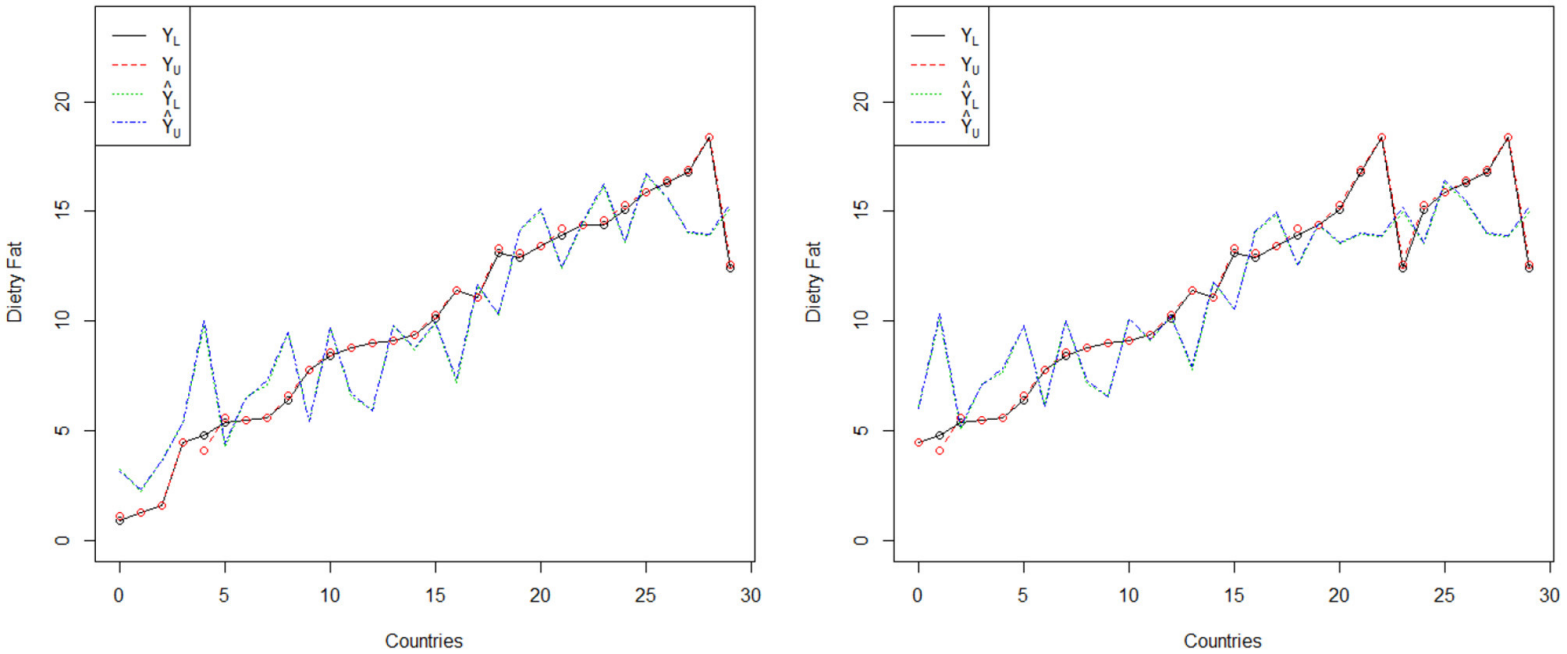
The trended lines of two regression models.

In this section, the comparison of the proposed regression model is given with the existing model proposed by Aslam and Albassam (14) in terms of trended values. The trended values of both models are presented in Table 3. The trended lines of both regression models are shown in Figure 1. From Figure 1, it can be noted that the trended values are close to actual values of prostate cancer for the proposed regression model. On the other hand, the trended values are away from the actual values of prostate cancer for the existing regression model proposed by Aslam and Albassam (14). From this comparative study, it can be concluded that the proposed model is quite suitable to apply for the forecasting of prostate cancer patients as compared to the existing regression model under the presence of uncertainty.

## Measures of Indeterminacy Based on Cancer Data

In this section, we will present the neutrosophic forms along with the measures of indeterminacy of the values of $\overset{n_{NT}}{\underset{i=1}{\sum }}{\left(Y_{NT}-{Ŷ}_{NT}\right)}^{2}$. The neutrosophic form of $\overset{n_{NT}}{\underset{i=1}{\sum }}{\left(Y_{NT}-{Ŷ}_{NT}\right)}^{2}$ of the proposed model can be expressed as: $\overset{n_{NT}}{\underset{i=1}{\sum }}{\left(Y_{NT}-{Ŷ}_{NT}\right)}^{2}=128.18+137.70I_{N}\epsilon \left[0,0.07\right]$. It means that the error sum of square under uncertainty can be from 128 to 137 with the measure of indeterminacy is being 0.07. The neutrosophic form Ŷ_*N*_ for example for country#4 can be given as: Ŷ_*NT*_ = 6.11−6.04*I*_*N*_ ϵ [0, 0.01]. From this neutrosophic form, the first value 6.11 indicates the trend values for the regression model under classical statistics. The second value 6.04*I*_*N*_ indicates the indeterminate part of the neutrosophic form. From this study, it can be noted that the death rate due to dietary fat will be from 6.04 to 6.11 per 100,000. The proposed regression model gives the trended values in intervals rather than the exact values. Therefore, the proposed model is reasonable to apply for the forecasting of the death rate due to dietary fat.

## Conclusions

In this paper, a new trimmed regression model under the neutrosophic was introduced. The mathematical model of the new regression model along with its neutrosophic form was given. The trimmed neutrosophic correlation was also introduced in the paper. The proposed trimmed regression is applied to prostate cancer. The efficiency of the proposed model is discussed with the existing regression model under neutrosophic regression. From the comparisons, it is found that the proposed model provides the minimum error sum of square as compared to the existing model. It is also concluded that the proposed model can be effectively used in forecasting prostate cancer as compared to the existing model. The proposed method can be applied in different areas of applications such as decision-making and multi-level programming. The proposed regression model can be used in medical science, business, and social science as future research.

## Data Availability Statement

The original contributions presented in the study are included in the article/supplementary material, further inquiries can be directed to the corresponding author/s.

## Author Contributions

MA and AA-M wrote the paper. Both authors contributed to the article and approved the submitted version.

## Funding

This work was supported by the Deanship of Scientific Research (DSR) at King Abdulaziz University, Jeddah. The authors, therefore, acknowledge with thanks DSR technical and support.

## Conflict of Interest

The authors declare that the research was conducted in the absence of any commercial or financial relationships that could be construed as a potential conflict of interest.

## Publisher's Note

All claims expressed in this article are solely those of the authors and do not necessarily represent those of their affiliated organizations, or those of the publisher, the editors and the reviewers. Any product that may be evaluated in this article, or claim that may be made by its manufacturer, is not guaranteed or endorsed by the publisher.
